# Dr. Coxe's Inquiry into the Claims of Dr. Harvey to the Discovery of the Circulation

**Published:** 1835-01-01

**Authors:** 


### An Inquiry into the Claims of Dr. W. Harvey to the Discovery of the Circulation of the Blood, &c. &c. &c. By *J. Redman Coxe*, M.D. &c. &c. &c. Philadelphia, 1834.

We admire the erudition, the elaborate research, and the unwearied industry of Dr. Coxe, as manifested in this volume; but we cannot extend the same admiration to his wisdom—nor yet to the *spirit* with which the inquiry is conducted. We know not how the matter may stand on the western shores of the Atlantic, but we are quite certain that not one in five hundred of the profession here care a rush whether Galen, Aristotle, Hippocrates, Cæsalpinus, and hundreds of others, anticipated our countryman by conjectures, hypotheses, or vague suppositions respecting the circulation of the blood. No great discovery was probably ever made instanter. Conjectures long precede proofs, in most instances. The real and effective discoverer, we imagine, is he who fixes the attention of the world on, and *proves* the discovery, by bringing it into complete operation. If Harvey or some other person had not *demonstrated* the circulation of the blood, all the hints and suppositions of his predecessors, from Hippocrates downwards, would have gone for nothing. Of what use was the *actual knowledge* of vaccination, possessed by the Gloucershire farmers, till Jenner fixed the attention of the profession on it, and proved its efficacy in preventing variola? Great numbers of Harvey's contemporaries denied the truth of the discovery—and afterwards, when the world acknowledged the truth of it, they attempted to prove that the circulation was known to many others before he was born. This has ever been the case, and arises from the envy and jealousy which men feel towards each other while living and rivals; but we did not expect to find such a man as Dr. Coxe raking up the musty records of antiquity for obscure passages that might deprive Harvey of a fame which has now been almost unanimously settled on his name for nearly two centuries. And for what purpose? Will Galen's ear be soothed, or Harvey's offended by the translation of laurels from the memory of one to that of the other? Alas no! Galen has more posthumous fame than the most ambitious could covet—and Harvey's claim to the discovery of the circulation will not be set aside by a hundred volumes written with all the diligence, learning, and scrutiny even of a Coxe. Should our author ask us if we had perused his arguments and the documents on which they are founded, we would candidly acknowledge that we did not read one-tenth part of them—and for this reason, that we think a great deal too much of precious time and unquestionable erudition has already been expended on the writing of the book—therefore will we not increase the loss by reading it. If the discovery of the circulation has not, as Dr. Coxe asserts, been of any material use in the practice of physic or surgery,* of what use can it be to shift the discovery itself from one man to another? Let Dr. Coxe answer that question.

"In this last particular, (says Dr. C.) its general influence may, perhaps, be judged of, by the following extract from Pitcairn; who shows, I apprehend, both with candour and with justice, how little, if at all, it superseded the practice of the ancients! At p. 17:

'Omnes vero medici, qui methodum ullam, quamvis circulationi, ut credi volunt, convenientem tradiderunt, *uno ore docent,* sanguinem, aut in partibus, aut glandulis hærere: et quia sanguis, sive crassior, sive subtilior, in partium interstitiis detentus, eadem omnia symptomata et inferre et pati potest, quæ veterum sanguis circulandi nescius. *Idcirco eadem medendi methodus a recentioribus est ubique fere adhibita, quæ antiquis placuit, quamvis plerumque experientiæ et legibus circulationis contraria. Unde non est mirandum non majorem factam esse mutationem in arte medica,* cum morbi plerique oriantur vitio circulationis in vasis minimis, quam multi recentiorum non melius Hippocrate et Galeno intelligere se demonstrant.'

These are strong, but perfectly just estimates, of the *very slight degree of real practical improvement,* beyond that of former experience, which Harvey's discovery of the circulation, admitting it to be both new and perfect, had actually introduced." 88.

Now it appears to us that Pitcairn, in the above passage, alluded exclusively to the practice of physic—"arte medica"—and that he could not be

---

* See page 88.

so blind as not to see the importance of a knowledge of the circulation in the practice of *surgery*. Without this knowledge, on which side of an aneurism would a surgeon cut, with the best chance of success? But it is needless to pursue the subject. The following is a fair specimen of Dr. Coxe's arguments and facts. Hippocrates, De alimento, speaks thus:—

"'Venarum radix hepar est: arteriarum radix est Cor. *Ex his per omnia* sanguis et spiritus *pervagatur*, calorque *per ea* permeat.' And could such expressions mean ought, or any thing, if not implying all that is now implied or understood by the Harveian route? Is not the blood distinctly characterized as flowing to every part, by this vascular apparatus, and the heat also; and is much more now implied, than was fully appreciated by Hippocrates; although not speaking exactly in similar terms; or denoting that particular route, which was more correctly laid down by Harvey, but without completing it? can this alone confer the exalted privilege awarded him? and shall not an iota of credit be allotted to others! Well may these neglected worthies, when viewing their birthright and blessing surreptitiously bestowed on a younger brother, like Esau to Isaac, exclaim, 'Hast thou but one blessing, O my father!' Can we draw no probability of an individual view of a particular subject, except it be clothed in one peculiar form of speech! Can the following extract by Pitcairn from Hippocrates 'De Corde,' admit of reasonable doubt as to a full conviction of a circulation, and that *perpetual;* although the precise route was then, and *is yet*, not conclusively settled! 'Hi sunt humanæ naturæ fontes; hinc flumina excurrunt, quibus corporis alveus irrigatur.' Surely the above, and others that might be adduced, might well establish a prior claim to the doctrine of a circulation." &c. 97.

When such crude absurdities, ignorance, and errors of the ancients, are put forth to disturb the merit of Harvey, as to the discovery of the circulation, we may well hope and believe that the wise "FATHER OF ALL" has placed an impenetrable barrier between the dead and the living—if it were for no other purpose than to "save us from our friends," when they are injudicious enough to hold forth our *failings* to the world as pre-eminent *virtues!* If Hippocrates can see, from the Heavens above, or the Hades below, the exertions of Dr. Coxe to prove his anticipations of Harvey, he will sigh from the inmost recesses of his soul, and bitterly lament that he ever ventured on speculations respecting the circulation of the blood, which were grounded on mere conjecture, and without an atom of proof—or, what is more important, without a shadow of truth.

Considering, then, the short and rounded period of man's existence in this world, the difficulties that beset him in acquiring medical knowledge, and the absolute necessity of dedicating every spare moment to *useful* science, we cannot help deploring the waste of time and talent which the work before us has cost. We have been, we believe, much longer in the exercise of our profession than Dr. Coxe—we have, perhaps, laboured as hard, and observed as carefully as he has—and yet we confess that the wide field of observation long open before us, has only tended to make us regret the little that we know, and the immensity we have yet to learn—without time to do so! How then, we ask Dr. Coxe, can he afford to throw away so many months and years upon researches that, confessedly, can tend to no useful practical purpose whatever?

Dr. Coxe deplores the little attention which is paid by the moderns to the writings of the ancient medical authors. There was a time when we had some leisure, and we took that opportunity of wading through the fathers of physic, from Hippocrates to Sydendam, marking, with the pencil, every passage that appeared either curious or useful. We have no hesitation in asserting that we derive not one iota of advantage from all this labour, when we come to the bedside of sickness—unless it be from *exercise* of the mind, which we strongly suspect might have been more beneficially exercised in other ways. We cannot, therefore, conscientiously advise our junior brethren to study the fathers of physic, till after they have acquired all the knowledge, immediate and collateral, which the moderns have accumulated. This done, we recommend them to dedicate nine-tenths of their time to the study of Nature, as exhibited in the phenomena of sickness and health,—and the other tenth to—Hippocrates and his descendants, if they please.

Although we cannot approve of the *animus* which Dr. Coxe occasionally displays towards Harvey, in his strictures and criticisms; yet we fully and freely acquit him of any "paltry desire to diminish the glories that have encircled his brow." We sincerely hope that the next offspring of Dr. Coxe's head and pen will call for more commendation in the pages of the MEDICO-CHIRURGICAL REVIEW.

